# Reassessing the approach to informed consent: the case of unrelated hematopoietic stem cell transplantation in adult thalassemia patients

**DOI:** 10.1186/1747-5341-9-13

**Published:** 2014-08-12

**Authors:** Salvatore Pisu, Giovanni Caocci, Ernesto d’Aloja, Fabio Efficace, Adriana Vacca, Eugenia Piras, Maria Grazia Orofino, Carmen Addari, Michela Pintor, Roberto Demontis, Federica Demuru, Maria Rita Pittau, Gary S Collins, Giorgio La Nasa

**Affiliations:** 1 Department of Public Health, Clinical and Molecular Medicine, University of Cagliari, Cagliari, Italy; 2 Mediterranean Center of Clinical Bioethics, University of Cagliari-Sardegna Ricerche, Cagliari, Italy; 3 Hematology, Department of Medical Sciences, University of Cagliari, Cagliari, Italy; 4 Bone Marrow Transplantation Center, Hospital “R. Binaghi”, Cagliari, Italy; 5 Health Outcome Research Unit, Gruppo Italiano Malattie Ematologiche dell’Adulto (GIMEMA), Data Centre, Rome, Italy; 6 Department of Biomedical Science and Biotechnology, Pediatric Clinic of the Bone Marrow Transplant Centre, University of Cagliari, Cagliari, Italy; 7 Centre for Statistics in Medicine, University of Oxford, Oxford, UK

**Keywords:** Patient-doctor relationship, Communication bias, Conscious consent

## Abstract

**Introduction:**

The informed consent process is the legal embodiment of the fundamental right of the individual to make decisions affecting his or her health., and the patient’s permission is a crucial form of respect of freedom and dignity, it becomes extremely important to enhance the patient’s understanding and recall of the information given by the physician. This statement acquires additional weight when the medical treatment proposed can potentially be detrimental or even fatal. This is the case of thalassemia patients pertaining to class 3 of the Pesaro classification where Allogenic hematopoietic stem cell transplantation (HSCT) remains the only potentially curative treatment. Unfortunately, this kind of intervention is burdened by an elevated transplantation-related mortality risk (TRM: all deaths considered related to transplantation), equal to 30% according to published reports. In thalassemia, the role of the patient in the informed consent process leading up to HSCT has not been fully investigated. This study investigated the hypothesis that information provided by physicians in the medical scenario of HSCT is not fully understood by patients and that misunderstanding and communication biases may affect the clinical decision-making process.

**Methods:**

A questionnaire was either mailed or given personally to 25 patients. A second questionnaire was administered to the 12 physicians attending the patients enrolled in this study. Descriptive statistics were used to evaluate the communication factors.

**Results:**

The results pointed out the difference between the risks communicated by physicians and the risks perceived by patients. Besides the study highlighted the mortality risk considered to be acceptable by patients and that considered to be acceptable by physicians.

**Conclusions:**

Several solutions have been suggested to reduce the gap between communicated and perceived data. A multi-disciplinary approach may possibly help to attenuate some aspects of communication bias. Several tools have also been proposed to fill or to attenuate the gap between communicated and perceived data. But the most important tool is the ability of the physician to comprehend the right place of conscious consent in the relationship with the patient.

## Introduction

There is worldwide agreement that principle of informed consent is intended to reflect the concept of patients’ autonomy and self-determination and to be the legal embodiment of the right of each individual to make decisions about his or her own health care [1]. In Italy, the legal basis for informed consent is well established by article 32 of the Italian Constitution that asserts that patients cannot be forced to submit to a medical treatment, unless the treatment is required by law. However, if the need to respect the patient’s autonomy and its contribution to the good of the patient are not well understood, this unquestionable right of patients cannot be properly respected. We contend that obligations arising from moral or legislative precepts do not adequately reflect the true value of informed consent.

The importance of informed consent is habitually stressed by several scholars referring to a quote of 1914 by Justice Cardozo that states: «Every human being of adult years and sound mind has a right to determine what shall be done with his own body; and a surgeon who performs an operation without his patient’s consent commits an assault, for which he is liable in damages» [2]. But a true interpretation of informed consent actually dates back as far as 1895, when George Surbled stated that «patients have the right to refuse any kind of trial or therapy merely barricading themselves behind their wish without any justifications. In fact they are the only masters of their life, then of their body that they can use as they like» [3]. This statement acquires additional weight when the proposed medical treatment may potentially be detrimental or even fatal. But what should inspire the concept of informed consent? By honor of their profession, physicians have the moral responsibility to identify reasonable treatment options for each patient and then to discuss with them the benefits and potential risks of these treatment options, answer their questions as openly and honestly as possible to identify the option they feel is most appropriate and finally to accept the patient’s choice (so long as they can do so in good conscience). The fundamental goal of this long procedure that consists in transmitting the information and obtaining informed consent, is to ensure that patients, are fully aware and conscious both of the natural threats of their disease and of the potential possibilities of recovery. The true essence of informed consent is to transfer the knowledge of the risks and benefits from the one who knows them (namely, the physician) to the one who has to go through and experience them (the patient). Since β-Thalassemia major is a disorder in which this challenge is particularly relevant, mutual understanding among physicians and patients is essential in this process.

### Thalassemia

β-thalassemia is a severe hereditary haemolytic anaemia that arises from the reduced or absent synthesis of the haemoglobin subunit beta. Clinical findings include severe anaemia, hepatic fibrosis and cirrhosis, diabetes mellitus, hypogonadism, growth retardation, sexual immaturity, moderate to severe pulmonary syndromes and cardiac disorders. Myocardial disease is by far the most important life-limiting complication and is responsible for about 70 percent of deaths in these patients [4].

Allogenic hematopoietic stem cell transplantation (HSCT) remains the only potentially curative treatment [5]. On the other hand, transfusion and iron chelation therapies are a valid alternative in patients who are compliant with treatment recommendations. In fact, the availability of better treatment strategies has considerably improved the survival and complication-free survival rates in these patients [6-8].

Unfortunately, unrelated HSCT in thalassemia patients pertaining to the class 3 of the Pesaro classification (mostly adult patients with presence of hepatomegaly, portal fibrosis and a history of irregular iron chelation) is burdened by a high transplantation-related mortality risk (TRM: all deaths that are considered to be associated with transplantation), equal to 30% according to published reports [9-11]. Graft versus Host Disease (GVHD), i.e. a reaction of donated bone marrow against the patient’s own tissue, is a major cause of post-transplantation morbidity and mortality but, although the use of new protocols has consistently reduced the risk of toxicity, the percentage of TRM is still high [12]. Hence, patients without an HLA-identical sibling donor are left with the difficult choice of whether they should continue traditional therapy, which can offer a life expectancy of fifteen years or more, or accept the high risk of unrelated HSCT in the hope of definitively eradicating the disease. This latter point marks the fundamental difference between thalassemia and other diseases, such as leukemia or myeloma, which are characterized by their unfavorable prognosis and the lack of an alternative to HSCT [13].

It is well known that in medicine it is mandatory for patients and physicians to thoroughly discuss the advantages, limits, and complications of the all so far available potentially effective therapeutic approaches. Indeed, several clinical reports have stressed the importance of communication strategies and of their introduction into routine medical practice [14,15]. This study combines the results of unrelated HSCT, performed in 34 adult class 3 thalassemia patients, with the main communication factors between patients and physicians. In thalassemia the role of the patient in the informed consent process leading up to HSCT has not been fully investigated so far.

We decided to evaluate whether communication biases, heuristics, distorted processes of recall and misunderstanding could affect the clinical decision-making process and compromise the informed consent process for unrelated HSCT. We also address the legal and deontological issues with particular emphasis on the physician-patient healing relationship and the moral aspects of clinical decision-making. Informing patients of all the factors involved in their health care is the primary responsibility of the physician who can thereby guarantee a more “self-aware” informed consent process to patients.

We hypothesized that the information provided by physicians in the medical scenario of HSCT might not be fully understood by patients and that misunderstanding and communication biases may affect the clinical decision-making process. Several errors in judgment may cause bias in communication between patients and physicians. Under a condition of uncertainty or in case of high risk, human beings are prone to take mental shortcuts commonly known as “heuristics”. Cognitive psychologists have categorized several heuristics: *availability* (tendency to judge an event by the ease with which relevant examples are recalled), *anchoring* (to use an initial piece of information to make subsequent judgments); *framing effects* (to reach a decision based on the *‘framework’* within which the information was given); *blind obedience* (to indiscriminately follow the decision of an authority) [16]. Heuristics are commonly used by patients within the informed consent process and they seem to be at least partly responsible for the discordance observed between the perceptions of patients and their treating physicians with regard to the risks and benefits of treatment [17].

## Methods

Thirty-four consecutive adult class 3 thalassemic patients (16 males and 18 females) aged more than 16 years (median 21, range 16 - 37) were transplanted from an unrelated donor after a myeloablative regimen in two different Italian bone marrow transplantation centers. Patients’ clinical features are shown in Table 1. Every informed consent session and all the information provided to patients were fully documented to get a realistic picture of the meeting. All the physicians involved collected and discussed in advance literature data on GVHD and mortality risk rates during a consensus conference. The analysis of the informed consent process was conducted on the 25 surviving patients (12 males and 13 females) after obtaining a written informed consent from them or from their parents in the case of children under the age of 18 years. A questionnaire was either emailed or given personally to patients. A second questionnaire was administered to the 12 physicians who treated the patients enrolled in this study. Written informed consent was provided by the patients according to the Declaration of Helsinki. The study was approved by the Ethics Board of the University of Cagliari, School of Medicine.

**Table 1:** Patient features

**Variable**	**Frequency**	**%**
**All patients**	21 (16-37)	
Median recipient age (years, range)		
Gender		
Male	16/34	47
Female	18/34	53
Death	9/34	27
Rejection	0	0
Grade II-IV acute GVHD (in dead pts)	6/9	67
Grade II-IV acute GVHD (in live pts)	3/25	12
Chronic GVHD	3/25	12
**Alive with graft**	**25/34**	**73**
Gender		
Male	12/25	48
Female	13/25	52
Median test age (years, range)	25 (18-35)	
≤ 20	13	52
≥ 20	12	48
School level at the time of HSCT		
High	14/25	56
Low	11/25	44

GVHD: Graft Versus Host Disease.

### Questionnaires

The questionnaires were developed in collaboration with a multidisciplinary team including an expert psychologist, surgeons and nurses. Physicians and patients were asked to recall the time when they respectively informed and were informed for the first time of the possibility of unrelated HSCT. The questionnaire that was administered both to physicians and patients contained the following 6 items, assessed with the visual 7 item Likert-type scale or percent values (Table 2): 1) the transplant-related mortality risk perception on a seven-item Likert-type scale; 2) the mortality risk rate communicated by the physician and recalled by the patients (this item was intended to detect whether there was a possible *frame effect* between numeric variables provided by the physicians and those recalled by the patients); 3) the percent value of mortality risk that was considered acceptable to undergo the transplant procedure; 4) the perception of GVHD as a life-threatening condition; 5) how much previous additional information (from family, friends with thalassemia, TV, internet etc.) had influenced the choice of undergoing HSCT (this item allowed to detect a potential *availability* heuristic); 6) the strength of the patient’s motivation to undergo HSCT before the informed consent session with the physician (this item allowed to detect an eventual *anchoring* heuristic).

**Table 2:** Communication factors explored among patients and physicians

	**Items of the questionnaire**
1	Transplant-related mortality risk perception (1 to 7 Likert Scale)
2	Percent value of mortality risk communicated and recalled (% value from 5% to 50%)
3	Percent value of mortality risk considered acceptable to undergo the transplant procedure (% value from 5% to 50%)
4	Perception of GVHD risk as a life-threatening condition (1 to 7 Likert Scale)
5	How much previous information (other patients, friends, TV, Internet) had influenced the patient before informed consent (1 to 7 Likert Scale)
6	Motivation to undergo HSCT before informed consent (1 to 7 Likert Scale)

GVHD: Graft Versus Host Disease.

The information was conveyed by the physician with the aid of numeric values such as a percentage or by showing Kaplan Meyer overall survival and TRM curves previously published in literature. In order to verify the results of the informed consent session over time, we wrote them all, e.g. failure and death rates, in a case history. These notes helped us compare the real rates the patients were told with the rates the patients remembered.

### Statistical analysis

Descriptive statistics were used to evaluate communication factors. The data we collected were summarized by reporting the proportion of patients answering each item. Association between patient preferences and demographical characteristics was evaluated by logistic regression analysis. Different groups were compared using the t-test for independent samples. Post-transplant survival probability was estimated using the Kaplan and Meier method [18].

## Results

### Clinical outcome

The Kaplan-Meier survival and TRM probabilities for the 34 patients studied were 73.5% and 26.5% respectively. Nine patients died of transplantation-related causes at a mean time of 170 days (range 17-470) after the HSCT procedure. Six of these patients presented acute GVHD (67%). Among the 25 surviving, transfusion-independent patients, 3 (12%) developed grade II-IV acute GVHD and 3 (12%) developed mild chronic GVHD. The median follow-up of surviving patients was 5.9 years (range 0.9-8.4).

### Questionnaires

The 25 surviving transfusion-independent patients returned the questionnaire after a median of 20 days, with answers to all the 6 items. The median time between HSCT and the survey was 12 months. The median age of patients at the time of the survey was 25 years (range 18-35). All the 12 treating physicians involved in the informed consent and in the clinical decision-making process completed the questionnaire. Table 3 shows the score differences between physicians and patients.

**Table 3:** Differences between patients and physicians perceptions

	**Physicians (N = 12) mean (SE)**	**Patients (N = 25) mean (SE)**	***p *** **value**
Transplant-related mortality risk perception	4,9	3.6	0,001*
(1 to 7 Likert Scale)	(0,3)	(0.2)	
Percent value of mortality risk communicated and recalled	30.0	20.4	0,005*
(% value from 5% to 50%)	(2.6)	(1.4)	
Percent value of mortality risk considered acceptable to undergo the transplant procedure	19.6	29.6	0,005*
(% value from 5% to 50%)	(2.5)	(2.2)	
Perception of GVHD as a severe life-threatening condition	5.4	4.1	0,006*
(1 to 7 Likert Scale)	(0.4)	(0.2)	
How much previous information had influenced the patient before informed consent	4.6	2.2	0,006*
(1 to 7 Likert Scale)	(0.4)	(0.3)	
Motivation to undergo HSCT before informed consent	4.6	4.8	**NS**
(1 to 7 Likert Scale)	(0.5)	(0.3)	

*Two-tailed t test significant value; GVHD: Graft Versus Host Disease; SE: standard error.

The mortality risk perceived by the patients, assessed on a 7-item Likert scale, was significantly lower if compared to the risk perceived by the physicians (3.6 vs 4.9, p = 0.001). The mean mortality risk rate that was actually communicated by the physicians was 30%, while the rate that was later recalled among the patients was significantly lower (20%). No demographic features influenced the patient’s recall of the mortality risk rate. Interestingly, the mortality risk that was considered to be acceptable by the physicians was significantly lower compared to the mortality risk that was considered to be acceptable by the patients (19.6 vs 29.6, p = 0.005). Among patients, this rate was significantly lower in the female patients than in the male ones (25% vs 34%, p = 0.003).

The perception of the risk of GVHD as a life-threatening condition (assessed on a 7-item Likert scale) was again significantly lower among the patients if compared to physicians (4.1 vs 5.4, p = 0.006). Female patients perceived a higher risk than male patients (5.2 vs 3.1, p = 0.024). The other demographic features that were analyzed such as the age or the level of education had no influence on the patient’s perception of severe GVHD as a life-threatening condition.

Finally, patients were strongly self-motivated to undergo HSCT before the informed consent process (4.8 on a 7 seven-item Likert-type scale). According to physicians’ evaluation, patients had been strongly influenced by information they had previously obtained from non-professional sources (4.6 on a 7 seven-item Likert-type scale).

## Discussion

The majority of therapies expose patients to some degree of risk. In fact, the beneficial effects of drugs and therapeutic approaches are always counterbalanced by undesired consequences. Therefore, it becomes essential for the physician and the patient to discuss beforehand the appropriateness of cure and/or treatment options, in order to achieve the patient’s best interest. (The) Physician must be able to balance beneficence and maleficence when considering the feasibility of treatment. Exploration of communication strategies between physicians and patients/relatives may lead to a better understanding of the factors that play a prominent role in the consent and in its comprehension. In general, patients who are satisfied with communication can cope with treatment-related stress better. Several authors emphasize the importance of imparting individually tailored information. In seeking consent, physicians must supply patients and/or participants with an adequate amount of information but, more importantly, they must make sure that the information has been fully understood [19,20]. The degree and the quality of information that should be disclosed to the patient represent a key question. A recent Cochrane review [21] identified ‘efforts by researchers to investigate interventions which seek to improve information delivery and consideration of information to enhance informed consent. although we were not able to say with confidence which types of interventions are preferable’. A common approach in the legal and bioethical community seems to consider either spontaneous, that is the information given spontaneously in spoken, written or video form and responsive disclosure, that comprises the information given in response to the patient’s questions [22]. In this model, widely adopted also in the Italian wards, a dual procedural aim is reached. The patient-physician relationship may rely on one side on the standard medical information a reasonable person may need to make the decision in question, while on the other hand the physician may enrich the information on the basis of the individual patient needs and doubts (in a tailored information protocol).

Although the number of patients involved in our study is robust enough to depict the entire phenomenon (25% of the Italian Pesaro class-3 transplanted patients with β-Thalassemia Major), (it) is not sufficient to draw a definitive conclusion. Despite this, we believe that our experience can be significant and instructive in the field of hematology to better understand the importance of informed consent in the patient-physician relationship.

The non-standardization and the non-psychometrical validation of the questionnaires used in the study is another potential limitation of our study. The Cochrane review [21] underlines that high levels of heterogeneity associated with many of the main analyses may encourage the meta-analysis results to be interpreted with caution and these limits are present in the vast majority of studies on informed consent. In fact, as we needed to assess our patients in a short time and no validated questionnaires allowing evaluation of the patients’ ability to recall the information provided during the informed consent process in this particular field of hematology were available, we decided not to undertake a validation process that would have required a long time.

Three issues of crucial importance need to be stressed at this point: 1 – the different way physicians and patients communicate and perceive risk; 2 – the different ways physicians and patients determine which level of risk is acceptable; 3 – the nonprofessional sources influencing the decision-making process.

### Discrepancy between doctors and patients

#### Communicated and perceived risks

The data obtained in this study confirmed a clear difference in the communication and in the perception of HSCT-related risks among the patients and the physicians involved in the clinical relationship. Patients recalled a mortality risk rate that was lower than the mortality risk rate they were actually told by the physicians (20% vs 30%). It is interesting to observe that female patients were more aware of the potential risks of the procedure. There may be several reasons for this miscomprehension. It is possible that during the informed process, patients felt anxious because of the uncertainty of the clinical outcome, and were therefore prone to anchor their choice to personal hope or to an initially positive impression. In fact, patients’ motivation to undergo HSCT before the discussion with the physician was very high (mean value of 4.6 on a 1-7 item Likert scale). Nevertheless, the questionnaires could not confirm a role for “availability heuristics” (mean value of 2.2 on a 1-7 item Likert scale). This discordance may be partly attributed to the way in which physicians assessed the benefits and risks of HSCT. Indeed, when physicians present HSCT in terms of chances of survival (positive frame), they paint a scenery that is psychologically much more acceptable than it would be if the procedure were proposed in terms of chances of death (negative trend). Ideally, a balance of both negative and positive views should be always presented [23]. Physicians not only have to improve their communication skills [24], but they also have to be sure that patients can remember in the best possible way the information provided during the consent process [25].

Patient’s recollection plays a fundamental role in the informed consent process: ordinary recollection processes may lead to distortions of crucial information, but even, patients may misremember information gained, due to different attitude toward the weighing information process before and after their decision. It has been shown that patients are likely to forget information based on *verbatim* memory (i.e. risks and complications rates), and to ground their choice on *gist* memory, which reflects enduring understanding and interpretation [26]. Unfortunately, informed consent is generally based on the communication of data, numbers and/or rates. In recent years, there has been a revolution in communication strategies. Manual information systems have increasingly been integrated by computer-based technologies capable of implementing the quality of communication. In particular, visual communication has been shown to be extremely effective in strengthening communication [23,27]: indeed, the majority of people seem to find it easier to remember pictures than words. However, these efforts were generally not so successful or showed mixed results in some patients [28]. Other strategies like corrected feedback, as Festinger et al suggest, may improve the recall of information, but the time interval required to realize this procedure is too long to be used in unrelated hematopoietic stem cell transplantation [29]. Surely, by improving our knowledge of the various communication strategies as visual graphs, movies, alternative formats to present and frame risk information- it may be possible to attenuate some of the current biases encountered within the decision-making process with the patient. However, we also have to investigate or assess the responsibility of the physician within the communication process, above all his/her understanding of the real role of informed consent in the relationship with the patient.

#### Acceptability of risk

Our results show that there is a significant difference comparing the acceptability of mortality risk in physicians and patients, i.e. 20% and 30% respectively, and comparing female and male patients, with the former being more realistic (34% vs. 25%). A higher mortality risk is likely to be accepted more easily by people with a serious and chronic disease than healthy individuals as the former have a stronger motivation to recover. This kind of reactions is not only comprehensible, but even acceptable. The way people react to illness was studied by Elisabeth Kubler Ross who indicated acceptance as the last stage and denial/refusal as the first one [30]. Diseases change the way people perceive illness and disease-related risks. In fact, if there is a real possibility of recovery, people suffering from a disease are disposed to accept high risks and severe side effects differently from healthy individuals. Therefore physicians have to be aware that patients perceive their disease and disease-related risks differently from them and that they should not conduct the informed consent process subjectively, but only focusing on scientific evidence.

#### Influences in decision making

Another intriguing item that emerged from our analysis concerns the moment at which patients take their therapeutic decision. Our results show that patients are strongly persuaded to undergo HSCT before starting the informed consent process. During the information process this event may undermine patient attentiveness to the information provided by physician, and undermine physicians motivation to provide detail if the physician believes that the patient has already made his/her decision. At a more in-depth analysis, a medical choice made by patients before they are provided with all the necessary information by their treating physician, albeit legal and acceptable threatens, the medical role of a ‘trusted guide on the illness journey’ [22]. The ratio of the information process relies on the need to bridge the knowledge gap between physician and patient, due to the cultural difference existing on the knowledge of medical issues. The main aim of the informed consent process is to give the patient all the scientific tools that are necessary to let him/her freely and deliberately decide for his/her own good. What if the physician is not longer identified (anymore) as the trusted scientific source of the ‘gold standard’ in information and treatment? Do physicians have a (the) duty to respect the patient’s therapeutic choice or refusal, even when such choices are not grounded in an accurate understanding of verified or validated assessment (their technical evaluation) of the available and shared medical protocols, as was the case in the recent Italian debate on FDA/EMA/AIFA unauthorized protocol (proposed by the Stamina Foundation) on the therapeutic use of stem cells? May naïve suggestions (or misinformation) coming from the web or from other questionable sources supplant a sound medical information process? In such scenario, a patient’s autonomy and a physician’s of beneficence both appear to be threatened and finding a shared decision making process becomes imperative.

### Consent: precious or unnecessary?

Our observations show that the consent process is not a mere bureaucratic procedure and that physicians should emphasize its importance more forcefully [31]. In fact, informed consent is sometimes considered as a bureaucratic burden or a legal myth [32], or even as an ineffective signing procedure [33], that regrettably continues to be used in a paternalistic way by some healthcare professionals [34]. Some physicians also use informed consent as a method of defensive medicine to protect themselves and not to preserve patients and their own dignity [35].

Conversely physicians should foster the patient-physician relationship and implement the informed consent process that entail respecting patients’ autonomy, acting for their good and empowering them to make decisions in line with their own values. On the other side patients’ decision may be grounded in unscientific and irrational basis even when the physician has provided them with accurate information.

All the issues that have been analyzed so far seem to cast doubts on the fundamental basis of the informed consent doctrine. A deep gap between the quality and the quantity of the clinical information provided by physicians and the one gathered and recalled by patients has been demonstrated. If these data are representative of a more general phenomenon, all the legal and ethical considerations on the fundamental basis of consent lose their importance. The clinical decision to undergo or to refuse the HSCT treatment is taken apart from the relevant issues concerning procedural security and personal values.

The validity of individual consent resides in the physician’s ability to convey exhaustive and essential information and, (contemporarily, depends upon) in the patient’s ability to properly interact with the doctor that will allow him/her to fully understand and recall the information provided. Before seeking the patient’s agreement, the physician has the fundamental duty to inform the patient completely and to make sure that he or she is aware of, and has genuinely understood, the risks and benefits both of the proposed treatment [36] and of alternative procedures and treatments. A new advance in the informed consent process can be approachable only in a new and right equilibrium between the pursuit of beneficence and reverence for autonomy.

### Beneficence, autonomy and self-aware consent

Over the last 50 years, patient(’s) freedom has become the hallmark of the patient-physician relationship, while beneficence has progressively lost the central position it had held in the past.

Precisely, the shift from beneficence to autonomy introduced a new approach to clinical decision-making that led to the doctrine of informed consent. Today, autonomy is widely considered to be one of the four master principles of bioethics and plays a prominent role in the medical setting. However, although the recognition of the value of autonomy has certainly been one of the most important achievements in the field of bioethics so far, autonomy and beneficence should not be considered separately as Beauchamp and Childress contend [37], or as contrasting principles, as Engelhardt asserts [38].

In our opinion, the concept of autonomy should not be considered as an independent principle and beneficence should not be identified with paternalism as Will maintained [31]. In fact, they are both cornerstones of the doctrine of informed consent, and beneficence cannot be achieved if patient’s autonomy is not respected.

The importance of autonomy is therefore closely related to the principle of beneficence. In fact, the good of the patient cannot be achieved if patients are not supported in their right to autonomous choice. Then, it is mandatory to consider autonomy as an unavoidable condition to attain the good and not a principle beside beneficence. Hence, beneficence and autonomy are inseparable concepts, but not inseparable principles, essentially because autonomy is not a principle [33]. Pellegrino and Thomasma successfully emphasized the principle of beneficence, but they have never challenged the idea of autonomy as a principle [39]. From our point of view, informed consent becomes an indisputable means to support autonomy, in order to achieve beneficence [40-42]. However, only beneficence can be recognized as a principle.

Consent was initially designed not only to preserve patients’ independence during the decision-making process [31], but also to allow physicians to act for the good of patients by promoting their understanding and, consequently, their right to choose or refuse the proposed treatment freely.

In fact, the good of patients cannot be achieved by a mere supply of information that may even be misleading in some cases. The term “informed consent” is itself so ambiguous that some authors have suggested to replace it with “Information for consensus”, as in the Italian deontological medical code [31]. However, this expression is still unclear, as it does not entail patients’ understanding during the consent process [43,44]. In fact, physicians often provide patients with a large amount of information, but they don’t verify whether patients understand the key points of the conversation.

This observation has led us to shift from the concept of “informed consent” to the more appropriate one of “self-aware consent” [45]. We are well aware of the debate around consciousness, that led several authors to stress the importance on the unconscious events arising from the patient-physician relationship. In particular, scholars who identify mental events with brain events are inclined to consider the process of legal consent as an illusion or, even, as a myth [46]. In this article we have already stressed the importance of unconscious events but, at the same time, we are deeply convinced that a self-aware consent can be obtained if all the factors involved are taken into account. Actually, the pursuit of self-aware consent is paramount. While, on the one hand, consent implies the concepts of information and contextualization, on the other, it is awareness that plays the most crucial role in our opinion. For this reason, physicians must not only pay attention to all the factors that are important to obtain this goal, but they also must identify the subconscious and unconscious barriers as much as possible. In our opinion, that is not a burden, but a fascinating task that helps physicians respect and promote patient’ autonomy when trying to reach beneficence. We believe that patients’ choice can be deliberately autonomous only if it is aware. In fact, autonomy and awareness are not correspondent: a decision could only apparently seem autonomous, and actually be not real aware.

We believe that the right way to build a correct procedure of consent must be searched in a sort of mediation between all of the factors involved in the relationship: the patient’s need to be respected in his/her autonomy and freedom and, at the same time, the doctor’s need to be recognized in his/her moral integrity and knowledge in the field.

The goal is to reach a real empowerment of the patient to be part of a shared-decision making model [47] in which ‘a patient may take some responsibility for her or his own health care rather than merely being loaded with a great deal of complex information’ [22]. In this context physicians could provide their patient with moral recommendations about his/her responsibility without becoming necessarily paternalistic.

We agree with Mazur who identifies at least six dimensions in the decision-making process that should be taken into account by the physicians involved in HSCT procedures: harms, benefits, scientific evidence, clinical experience, estimation experience and psychological experience involving estimates [48]. In our opinion, another dimension, i.e. self-aware consent, should be added. Self-aware consent is a valid procedure only at the time that the patient is giving consent; in fact, if some conditions are going to change, i.e. clinical picture, environmental conditions, consent must be reconsidered. Indeed, consent is somehow a dynamic procedure that portrays a specific situation at an established moment rather than a static process given once and for all.

## Conclusions

Self-aware consent is crucial in the patient-physician relationship and its achievement can be affected by several dilemmas. A multi-disciplinary approach (medical, psychological and ethical) may possibly help to smooth some of the challenging issues characterizing the information procedure. Several ways have been suggested to fill or attenuate the gap between communicated and perceived information, but physicians’ ability to value the informed consent within their relationship with patients undoubtedly plays a key role [21]. The pursuit of self-aware consent is paramount and physicians have to realize that respecting patients’ freedom is part of the goal of their profession and that self-aware consent in a shared-decision making model rather than informed consent is the only ground where freedom can be respected.

## Abbreviations

HSCT: Allogenic Hematopoietic Stem Cell Transplantation; GVHD: Graft Versus Host Disease; TRM: Transplantation-Related Mortality.

## Competing interests

The authors declare that they have no competing interests.

## Authors’ contributions

GLN and GC designed and coordinated the study, interpreted data and wrote the first draft; FE and GSC coordinated data analysis and interpretation; AV, EP, RL, MGO and CA recruited patients and administered survey instruments; ED addressed the ethical and legal aspects and helped draft the manuscript; RD, MP and MRP addressed the legal aspects; FD addressed the ethical aspects; SP addressed the ethical aspects and wrote the last version of the manuscript. All authors read and approved the final manuscript.
